# Insulin-like growth factor I and its binding protein-3 are regulators of lactation and maternal responsiveness

**DOI:** 10.1038/s41598-017-03645-5

**Published:** 2017-06-13

**Authors:** András H. Lékó, Melinda Cservenák, Éva Rebeka Szabó, János Hanics, Alán Alpár, Árpád Dobolyi

**Affiliations:** 1 Laboratory of Neuromorphology, Department of Anatomy, Histology and Embryology, Semmelweis University, Budapest, 1094 Hungary; 2 MTA-ELTE NAP B Laboratory of Molecular and Systems Neurobiology, Hungarian Academy of Sciences and Eötvös Loránd University, Budapest, 1117 Hungary; 3 MTA-SE NAP B Research Group of Experimental Neuroanatomy and Developmental Biology, Hungarian Academy of Sciences, Budapest, Hungary; 4 Department of Anatomy, Histology and Embryology, Semmelweis University, Budapest, 1094 Hungary; 5 MTA-ELTE Laboratory of Molecular and Systems Neurobiology, Department of Physiology and Neurobiology, Hungarian Academy of Sciences and Eötvös Loránd University, Budapest, 1117 Hungary

## Abstract

Adaptation to motherhood includes maternal behaviour and lactation during the postpartum period. The major organizing centres of maternal behaviour and lactation are located in the hypothalamic medial preoptic area (MPOA) and the arcuate nucleus, respectively. Insulin-like growth factor I (IGF-I) is an effector of the growth hormone axis; however, its function in the brain is largely unexplored. We identified increased maternal IGF binding protein-3 (IGFBP-3) expression in preoptic rat microarray data and confirmed it by RT-PCR. *In situ* hybridization histochemistry showed markedly elevated IGFBP-3 expression in the MPOA and the arcuate nucleus in rat dams. Prolonged intracerebroventricular injection of IGF-I or antagonism of brain IGFBP-3 with an inhibitor (NBI-31772) using osmotic minipumps increased pup retrieval time, suggesting reduced maternal motivation. Suckling-induced prolactin release and pup weight gain were also suppressed by IGF-I, suggesting reduced lactation. In addition, IGF-I-induced tyrosine hydroxylase expression and its specific phosphorylation in tuberoinfundibular dopaminergic neurons suppress prolactin secretion. Thus, IGF-I may inhibit both behavioural and lactational alterations in mothers. Neurons in the MPOA and arcuate nuclei express IGFBP-3 during the postpartum period to neutralize IGF-I effects. IGFBP-3 can prevent the blockade of maternal behaviour and lactation exerted by IGF-I, suggesting a novel modulatory mechanism underlying the behavioural and hormonal effects during central maternal adaptations.

## Introduction

Postpartum physiological and behavioural changes are important parts of mammalian reproduction, and they can be investigated using the rat as an animal model. Non-maternal females do not care about pups or even attack them, while mothers demonstrate well-defined maternal behaviours, e.g., nest building, pup retrieval to the nest, nursing, and decreased anxiety, in addition to lactation. These marked changes are the consequences of maternal adaptation of the central nervous system. Lactation is known to be driven by prolactin secreted from the pituitary^1^. Prolactin secretion is controlled by the inhibitory effect of dopamine produced by the tuberoinfundibular dopamine (TIDA) neurons. These neurons are located in the arcuate nucleus and project to the external zone of the median eminence and excrete dopamine into the pituitary portal blood vessels^2^. The modulators of prolactin release are known to affect the TIDA neurons; however, the major intrinsic regulators of the switch in the mode of these neurons, which permits the dramatically increased prolactin secretion required for lactation, need to be elucidated^2, 3^.

Prolactin and other hormones contribute to the initiation of maternal behaviours^4, 5^ but are not required for it^6, 7^. Rather, maternal behaviour is controlled by a complex neuronal network^8, 9^ in which the medial preoptic area (MPOA) plays a central role^10^. The density of active neurons is dramatically induced in the MPOA of parenting females^9^. Furthermore, lesions of the MPOA abolish the nest building and retrieving components of maternal behaviour in lactating females^11–13^, while electrical and optogenetic stimulation of this area enhances maternal responsiveness^8, 9, 14^. Although the molecular mechanisms of maternal motivation are unknown, gene expression alterations have been hypothesized to be involved^15^. Previously, we carried out a microarray study of the preoptic area and identified amylin as a maternally induced neuropeptide^16^. We validated and functionally characterized amylin in mother rats^17^ but did not evaluate other genes with altered mRNA expression. In the present study, we identified significantly altered genes in our previous microarray study^16^, compared them with previous microarray data^18^, and investigated the maternal function of a gene altered in both studies, insulin-like growth factor binding protein-3 (IGFBP-3).

Insulin-like growth factor binding protein-3 (IGFBP-3) binds insulin-like growth factor-I (IGF-I) in the plasma and extracellular space. Although 6 other IGFBPs exist, IGFBP-3 is the major carrier of IGF and binds the majority of IGF-I in circulation^19^. By binding IGF with high affinity in a functionally inactive complex, IGFBP-3 can inhibit the effects of IGF-I. IGFBP-3 overexpressing mice show intrauterine and postnatal growth retardation, confirming the IGF-neutralizing role of IGFBP-3^20^. In addition, IGFBP-3 may also have IGF-independent effects^21–23^. IGFBP-3 is normally expressed in the adult central nervous system (CNS) at a low level, mainly in non-neuronal cells, and the effects of IGFBP-3 on the CNS are largely unknown^24–28^.

IGF-I is mainly produced by the liver and is related to the growth hormone axis. IGF-I is released into the circulation and can reach the central nervous system via the blood-brain-barrier and blood-cerebrospinal fluid barrier. In addition, IGF-I is also expressed in the brain. The actions of IGF-1 are mediated by a cell surface receptor, type 1 IGF receptor (IGF-1R), which is the major transducer of IGF signals^29^. IGF-1 signalling in the brain supports neuronal survival and neuroprotection during the development of the CNS and in adults following brain injuries^30^. In the hypothalamus, IGF-I can feedback to the growth hormone axis but may also affect reproductive neuroendocrine functions^31–33^. However, the effects of IGF-I on lactation and prolactin release, an important aspect of reproduction, have not been investigated.

In this study, we first established that the expression of IGFBP-3 was significantly elevated in lactating rat mothers compared with that in mothers whose pups were taken away right after parturition (pup-deprived mothers). Second, we revealed the involvement of IGFBP-3 in the control of maternal behaviour with an IGFBP-3 antagonist. We also showed that the effect of IGF-I treatment was similar that of the IGFBP-3 antagonist. Third, we described the expression pattern of IGFBP-3 in the hypothalamus and identified two regions, the medial preoptic area and the arcuate nucleus, where its expression was markedly elevated in rat dams. Then, we demonstrated that the IGF-IGFBP-3 system regulates suckling-induced prolactin release. Furthermore, we identified the underlying mechanisms of the effects of IGF-I on prolactin-regulating tuberoinfundibular dopaminergic neurons both *in vivo* and *in vitro*.

## Results

### Genes with altered mRNA expression in the preoptic area of rat dams

To determine the genes that show altered expression in mothers, we re-evaluated our previous microarray study in the preoptic area of mother rats^16^ and compared the results of this analysis with a recent microarray study^18^. We identified 21 genes with highly significant differences in expression levels between the maternal and non-maternal groups (p < 0.007) in our previous experiment (Table 1). Islet amyloid polypeptide (amylin) exhibited the greatest elevation in mothers compared with that in pup-deprived controls and has been identified as a novel neuropeptide with maternal functions in the rat^17^. The expression levels of 5 other genes (ELL-associated factor 2, dopamine receptor 4, insulin-like growth factor binding protein 3, follistatin, and selectin) were 2–4 times higher in rat dams than those in the pup-deprived control group. Of these genes, insulin-like growth factor binding protein-3 (IGFBP-3) was also up-regulated in the study of *Driessen et al. 2014*. Therefore, we choose to further investigate the maternal function of this protein.

**Table 1:** Microarray data from lactating mother rats compared to non-maternal previous mothers whose pups were taken away right after delivery (pup-deprived mothers). The listed 21 genes showed highly significant (p < 0.007) difference between the two groups in our microarray study (n = 4 rat for each group)^16^.

p	Fold change	Name of gene product	RefSeq	UniGene code
0.00001	25.732	islet amyloid polypeptide	NM_012586	Rn.11394
0.00015	0.466	similar to RNA binding motif protein 3	XM_001063211	Rn.18057
0.00016	0.422	aldo-keto reductase family 1, member B7	NM_053781	Rn.32702
0.00031	0.469	S100 calcium-binding protein A4	NM_012618	Rn.504
0.00034	0.442	microtubule-associated protein tau	NM_017212	Rn.2455
0.00045	0.415	similar to macrophage scavenger receptor 2	XM_001067252	Rn.76819
0.00050	0.438	procollagen, type III, alpha 1	NM_032085	Rn.3247
0.00052	0.465	similar to aldo-keto reductase family 1, member C19	XM_001062695	Rn.16371
0.00052	0.392	similar to ectonucleotide pyrophosphatase/phosphodiesterase 6	XM_001056025	Rn.8484
0.00059	0.359	KiSS-1 metastasis-suppressor	NM_181692	Rn.66008
0.00060	0.490	hypothetical protein	XM_001066258	Rn.139226
0.00069	0.367	calbindin 3, (vitamin D-dependent calcium binding protein)	NM_012521	Rn.9974
0.00084	0.373	asialoglycoprotein receptor 1 (hepatic lectin)	NM_012503	Rn.44300
0.00144	0.401	similar to procollagen, type I, alpha 1	XM_001081230	Rn.2953
0.00200	2.653	ELL associated factor 2	NM_172047	Rn.20681
0.00207	3.720	dopamine receptor 4	NM_012944	Rn.10159
0.00277	0.415	similar to Paired mesoderm homeobox protein 2 (PRX-2) (Paired-related homeobox protein 2)	XM_001079701	Rn.93004
**0.00281**	**2.186**	**insulin-like growth factor binding protein 3**	**NM_012588**	**Rn.26369**
0.00537	2.473	follistatin	NM_012561	Rn.162557
0.00546	0.439	ring finger protein 141	NM_001001800	Rn.127990
0.00685	2.152	selectin, endothelial cell	NM_138879	Rn.10359

### RT-PCR and *in situ* hybridization histochemistry validation of the induction of IGFBP-3 in the preoptic area of rat mothers

**Figure 1:**
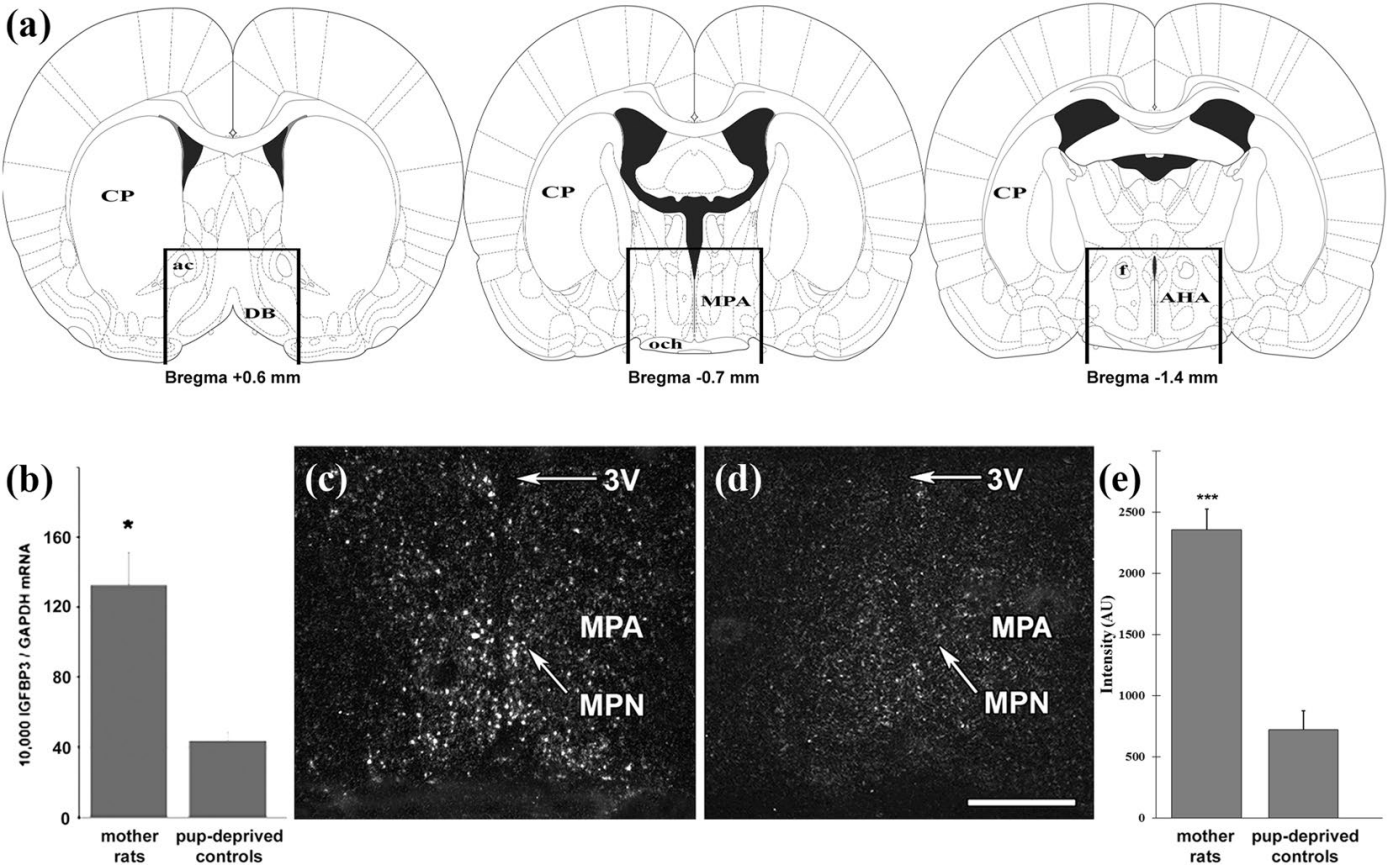
Induction of IGFBP-3 in the preoptic area of lactating rat mothers. (**a**) Schematic figures show the dissected area, which was used for RT-PCR validation of the microarray data. The rostral and caudal borders of the dissected area were at +0.6 and −1.4 mm from the bregma, respectively. A dorsal horizontal cut was made immediately above the level of the anterior commissure, and the lateral borders were cut 2 mm lateral to the midline. (**b**) IGFBP-3 mRNA level was 3 times higher in the preoptic area of rat dams (n = 9) compared with that in the preoptic area of pup-deprived controls (n = 8; *: p < 0.05). Data are presented as the ratio to GAPDH mRNA levels as the mean values ± SEM. (**c,d**) Dark-field photomicrographs of IGFBP-3 *in situ* hybridization histochemistry show that the IGFBP-3 mRNA hybridization signal (white) was elevated in rat dams (**c**) compared with that in pup-deprived rat mothers (**d**) in the medial preoptic area (MPA) and the medial preoptic nucleus (MPN). (**e**) Densitometric analysis shows that the IGFBP-3 labelling intensity is significantly elevated in the MPA of lactating mothers compared with that in pup-deprived controls (n = 6–6; ***p < 0.001, p = 0.00002). Density is expressed in arbitrary units (AU). The results are presented as the mean values ± SEM. Further abbreviations: 3 V – third ventricle; ac – anterior commissure; AHA – anterior hypothalamic area; CP – caudate putamen; DB – diagonal band; f – fornix; och – optic chiasm. Scale bar = 400 µm.

Preoptic samples were dissected from 9 lactating and 8 pup-deprived females for RT-PCR validation of the microarray results (Fig. 1a). We revealed higher IGFBP-3 mRNA levels in the preoptic area of lactating dams compared with those in mothers deprived of their pups (Fig. 1b) while there was no difference in the level of GAPDH mRNA between the 2 groups. We used IGFBP-3 *in situ* hybridization histochemistry to determine its distribution in the preoptic area (Fig. 1c,d). We used two different, non-overlapping *in situ* hybridization probes, which showed the same distribution pattern of IGFBP-3 expression and confirmed their specificity. The hybridization signal was present in the medial preoptic area, especially in the medial preoptic nucleus. The quantification of the autoradiography signal revealed an elevated level of IGFBP-3 in lactating mother rats (Fig. 1e).

### Antagonism of IGFBP-3 and IGF-I suppresses pup retrieval behaviour

**Figure 2:**
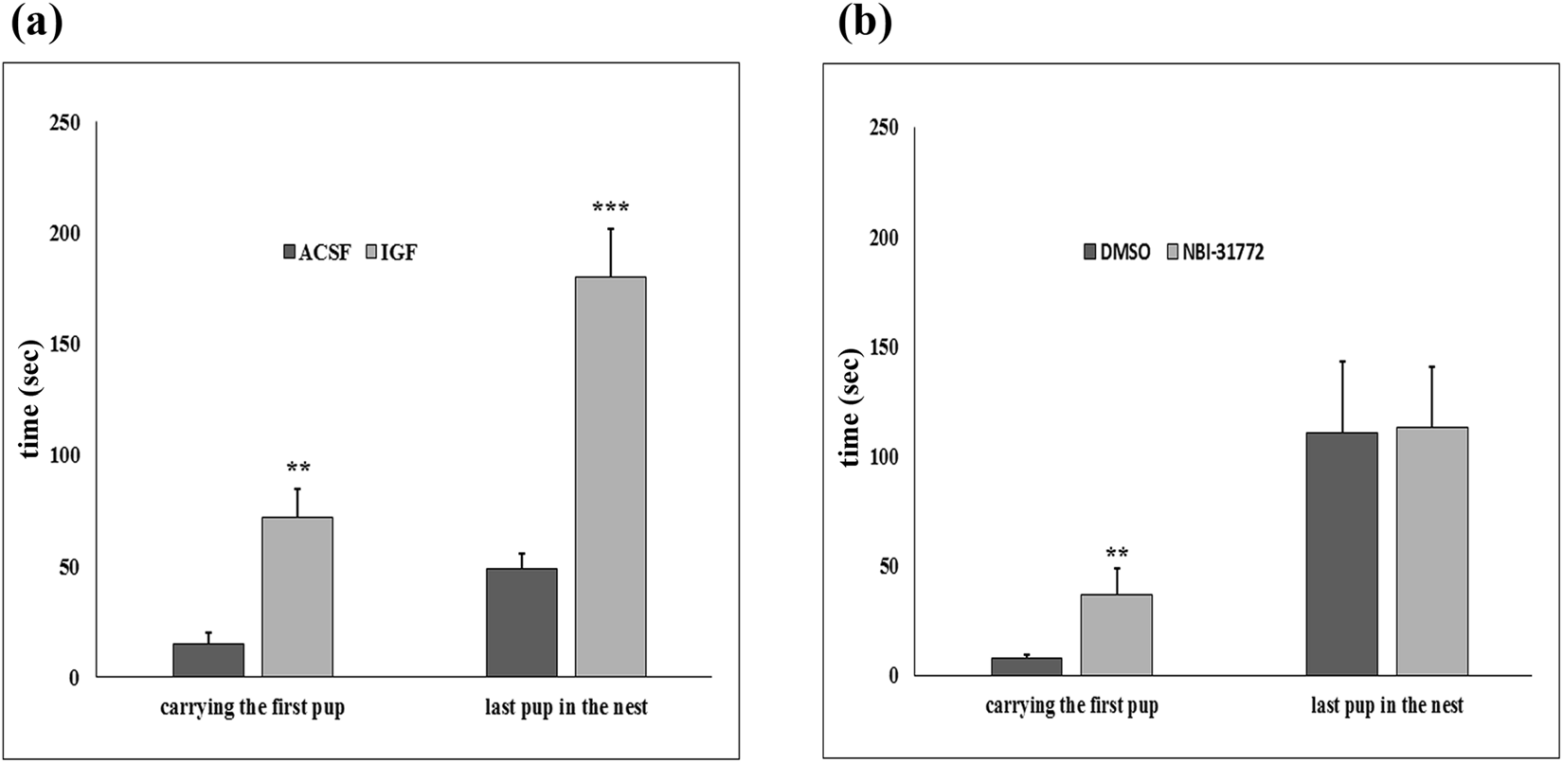
Antagonism of IGFBP-3 and IGF-I administration markedly lengthens the latency to retrieve pups. (**a,b**) Mothers were treated from the 2^nd^ postpartum day intracerebroventricularly (i.c.v.) via osmotic minipumps connected with brain infusion kits with artificial cerebrospinal fluid (ACSF) (n = 10) or dimethyl sulfoxide (DMSO) (n = 8) dissolved in ACSF as controls and with a nonpeptide IGFBP-3 ligand inhibitor, NBI-31772 (1.66 µg/µl in DMSO ACSF; n = 8) or IGF-I (4 µg/µl in ACSF; n = 10). Both treatments significantly increased the time to carry the first pup back to the nest (**p < 0.01, p = 0.0017 and 0.0037, respectively). IGF-I administration also increases the time to retrieve the last pup (***p < 0.001, p = 0.0008). The results are presented as the mean values ± SEM.

To assess the involvement of IGFBP-3 in maternal motivation, IGF-I and NBI-31772, a non-peptide ligand inhibitor of IGFBP-3, were continuously administered intracerebroventricularly (i.c.v.) via osmotic minipumps implanted in rat mothers on the 2^nd^ postpartum day. The first group of mothers was treated with IGF-I, while their control group (n = 10) received ACSF. Then, NBI-31772 dissolved in 1% dimethyl sulfoxide (DMSO) in ACSF was administered i.c.v. to another group. Their control group received 1% DMSO in ACSF i.c.v. We performed a pup-retrieval test on the 6^th^ day postpartum and found a markedly elevated pup retrieval time in the IGF-I- and NBI-31772-treated groups of mothers (Fig. 2a,b). These mothers took 4–5 more times to carry the first pup back to the nest. The time to retrieve the last pup was 3.7 times longer for the IGF-treated mothers than that for the ACSF group, but there was no difference between NBI-31772 and its control group. Starting on the 4^th^ postpartum day, we also investigated 5 elements of undisturbed maternal behaviour (high kyphosis, licking/grooming, prone nursing, supine nursing, mother out of the nest) for 5 days, 3 × 1 hour/day, 20 observations/hour and did not find any significant difference between the groups (Table 2). To examine the motility and anxiety of the mothers, we carried out an elevated plus maze test on the 7^th^ postpartum day (Table 2). We did not find any difference between the groups in the total number of entries, a marker of motility. IGFBP-3 antagonism and IGF-I administration did not cause changes in the open arm entry percentage, a marker of anxiety.

**Table 2:** Antagonism of IGFBP-3 does not change undisturbed maternal behaviour and elevated plus-maze test performance of rat dams. The ratio of 5 examined elements of maternal behaviours is shown in the table. In the elevated plus maze test, unchanged total number of entries suggest unaltered motility of the animals while the lack of significantly changed open arm entry frequency suggest unaltered anxiety-like behaviour.

	ACSF	IGF	DMSO	NBI-31772
total number of entries	10.57 ± 2.01	11.4 ± 1.65	12 ± 1.78	11 ± 1.58
open arm entries (%)	46.33 ± 3.24	36.36 ± 6	46.66 ± 4.51	45.22 ± 5.07
high kyphosis (%)	26 ± 2.34	21.43 ± 2.22	23.8 ± 1.98	23 ± 2.68
licking/grooming (%)	7.03 ± 0.54	5.13 ± 0.73	6 ± 0.84	7.96 ± 1.43
prone nursing (%)	21.14 ± 8.15	21.71 ± 7.91	24.62 ± 15.46	20.22 ± 5.24
supine nursing (%)	5.23 ± 4.17	7.17 ± 5.4	7.76 ± 7.91	6.8 ± 4.56
out of the nest (%)	42 ± 3.08	45.71 ± 3.1	39 ± 5.08	44.9 ± 4.72

### IGFBP-3 mRNA expression is markedly elevated in the arcuate nucleus of lactating rat mothers

**Figure 3:**
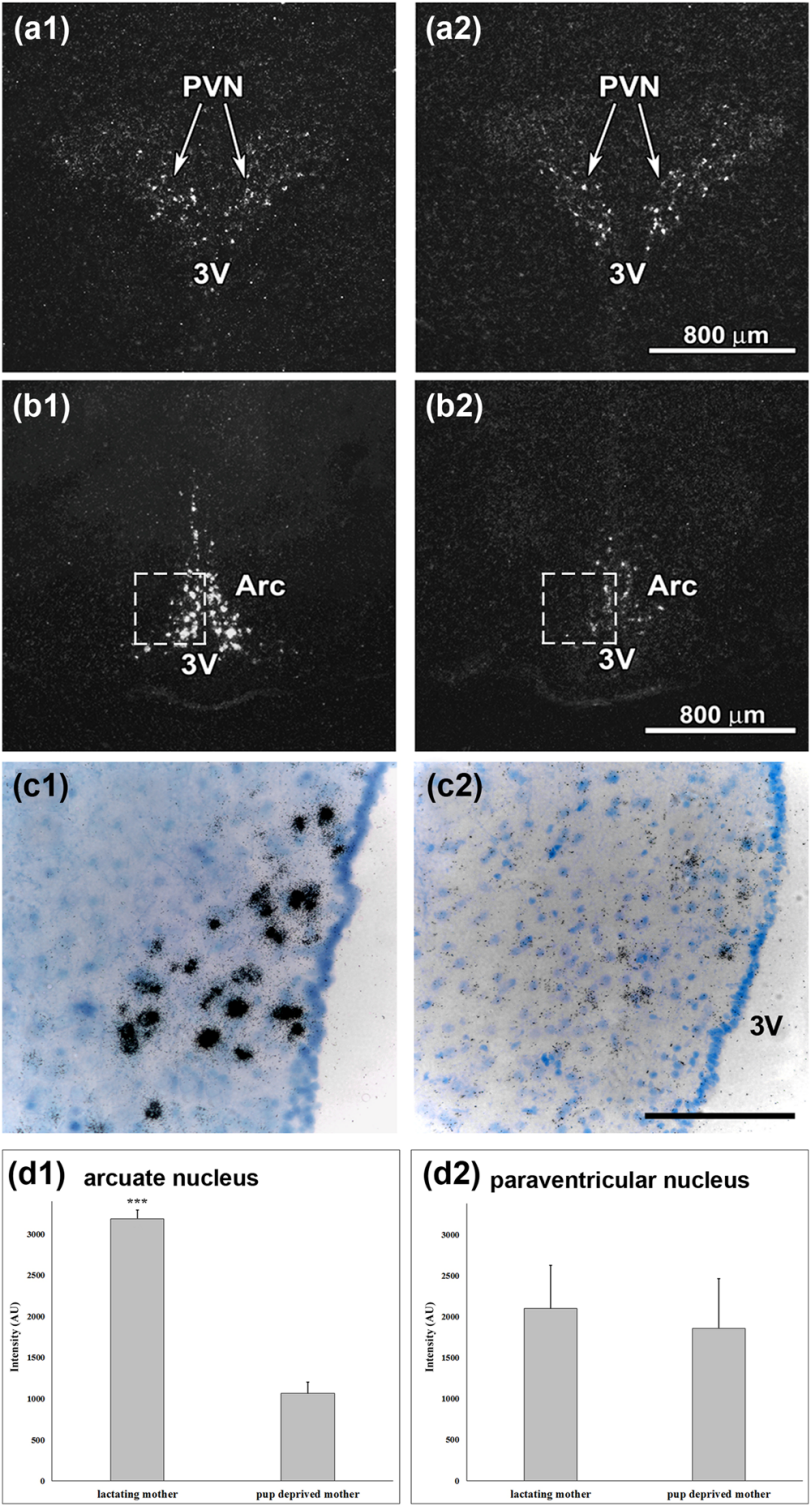
IGFBP-3 mRNA expression is present in the hypothalamus. (**a,b**) Dark-field photomicrographs of IGFBP-3 *in situ* hybridization histochemistry show that the IGFBP-3 mRNA hybridization signal (white) is present in the hypothalamic paraventricular nucleus (PVN) and the arcuate nucleus. In the PVN, the signal intensity appears similar between mothers (a1) and pup-deprived controls (a2). In contrast, IGFBP-3 expression seems more abundant in lactating mothers (b1) than in pup-deprived rat mothers (b2) in the arcuate nucleus (**c**) High magnification bright-field photomicrographs show the corresponding framed areas in b to demonstrate the increased number of individual autoradiography grains (black dots) in the dorsomedial subdivision of the arcuate nucleus in lactating mothers. (d1) Densitometric analysis reveals that the IGFBP-3 labelling intensity is significantly elevated in the arcuate nucleus of lactating mothers (***p < 0.001). (d2) There were no significant differences between lactating and pup-deprived females in the PVN. Density is expressed in arbitrary units (AU). The results are presented as the mean values ± SEM. Further abbreviations: Arc – arcuate nucleus, 3 V – third ventricle. Scale bars = 800 µm in (**a**,**b**) and 200 µm in (**c**).

*In situ* hybridization histochemistry revealed that the expression of IGFBP-3 is not confined to the preoptic area within the hypothalamus; the dorsomedial subdivision of the arcuate nucleus and the paraventricular hypothalamic nucleus (PVN) also contained IGFBP-3 mRNA (Fig. 3a–c). The quantification of the *in situ* hybridization histochemistry signal revealed that IGFBP-3 had an approximately 3-fold increase in the expression level in the dorsomedial subdivision of the arcuate nucleus in mother rats compared with that in pup-deprived controls (n = 6 − 6; Fig. 3d1). In contrast, there was no maternal increase in the expression level of IGFBP-3 in the PVN.

### IGFBP-3 is expressed in tuberoinfundibular dopaminergic neurons in the arcuate nucleus of lactating mother rats

**Figure 4:**
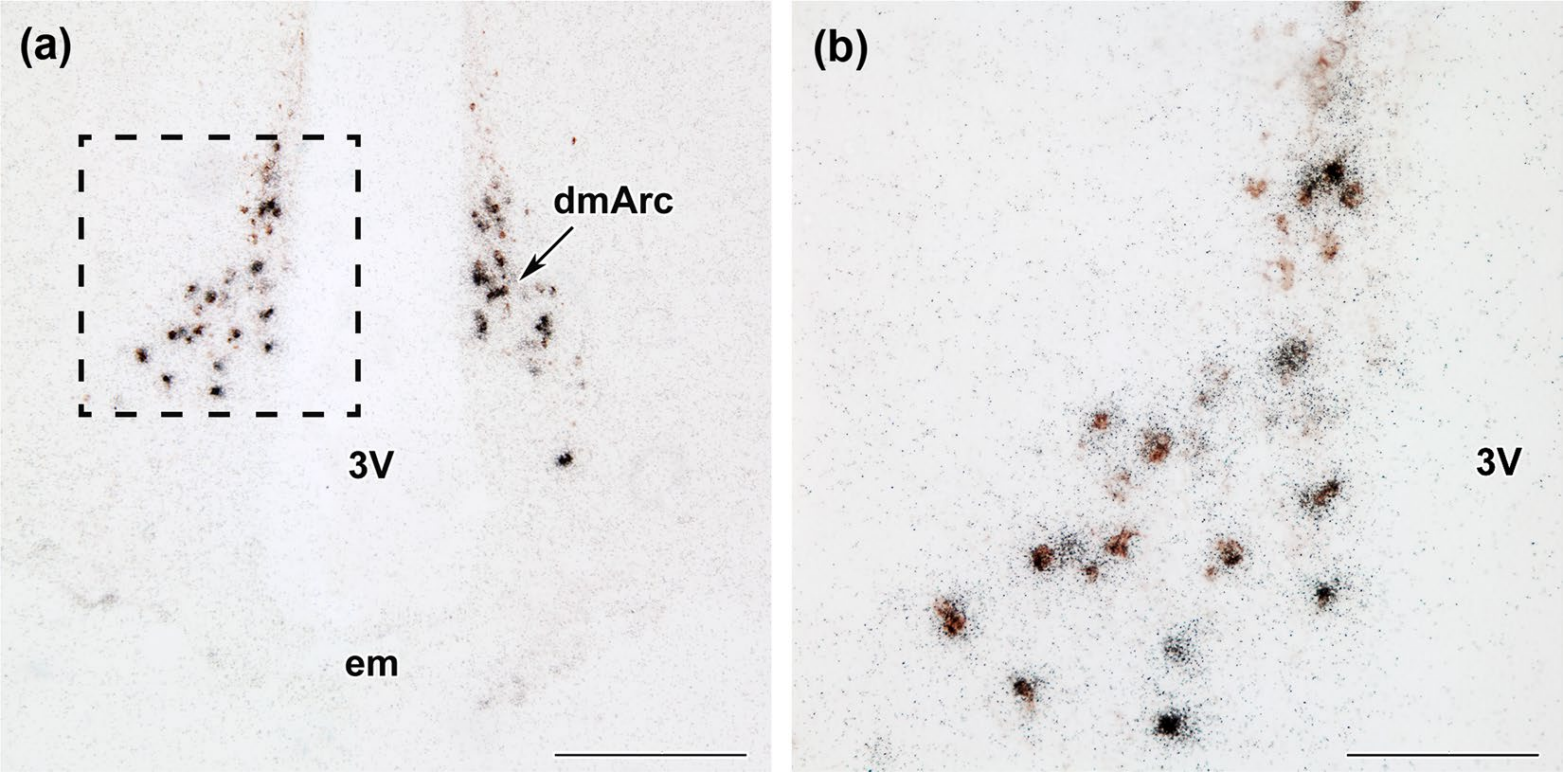
IGFBP-3 expression co-localizes with tyrosine-hydroxylase (TH) in the arcuate nucleus. The combination of IGFBP-3 *in situ* hybridization histochemistry (black dots) and TH immunohistochemistry (brown cells) shows that TH-positive cells in the dorsomedial subdivision of the arcuate nucleus express IGFBP-3 mRNA in lactating rat dams. Abbreviations include 3 V – third ventricle, dmArc – dorsomedial arcuate nuclei, em – median eminence. Scale bar = 400 µm for A, and 100 µm for B.

The location of IGFBP-3-expressing neurons was similar to that of dopaminergic neurons in the dorsomedial subdivision of the arcuate nucleus in lactating dams. Tyrosine hydroxylase (TH) is the main immunohistochemical marker of dopaminergic neurons in the arcuate nucleus, which regulates the secretion of prolactin from the pituitary. Therefore, we combined IGFBP-3 *in situ* hybridization histochemistry with TH immunohistochemistry to determine if dopaminergic neurons express IGFBP-3 mRNA in the arcuate nucleus (Fig. 4). We found a high degree of co-localization; 67.3% of tuberoinfundibular dopaminergic (TIDA) neurons contained IGFBP-3 and 85.9% of IGFBP-3 neurons contained TH immunoreactivity, suggesting that in the arcuate nucleus of lactating mothers (n = 5), IGFBP-3 mRNA expression is markedly elevated in TH-immunoreactive dopaminergic neurons.

### IGF-I lowers suckling-induced prolactin release

**Figure 5:**
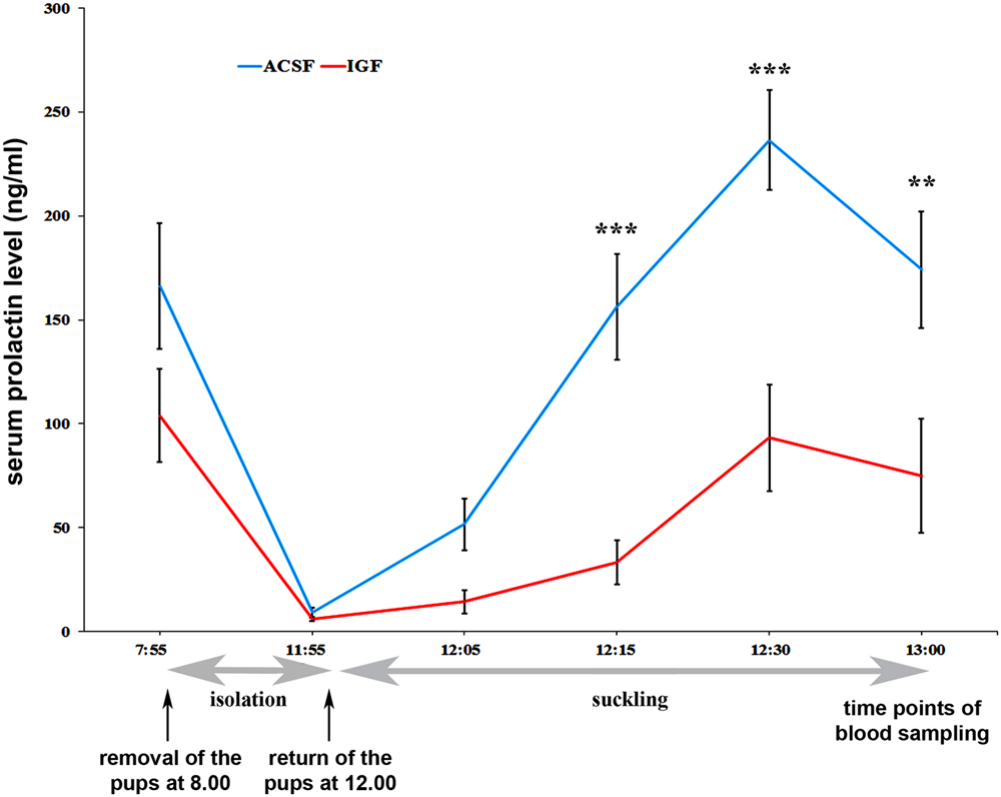
IGF-I administration significantly suppresses suckling-induced prolactin release. Blood was taken first at 8.00 AM, before the removal of the pups from their mother; second, right before the pups were returned after a 4-hour isolation period; the 3^rd^, 4^th^, 5^th^ and 6^th^ blood samples were obtained at 5, 15, 30 and 60 min after the pups were returned (start of suckling). The diagram shows the serum prolactin levels in mothers during the pup isolation-suckling protocol. The animals treated with IGF-I are shown in red (n = 7), while the ACSF-treated rats are shown in blue (n = 8). The x-axis shows time in a nonlinear fashion. Below the x-axis, the periods of pup separation and suckling are indicated with grey arrows. During the initial time point, before the isolation period, the plasma prolactin levels in the two groups of animals did not differ significantly as determined by Newman-Keuls post hoc test, even though a clear trend is visible for reduced levels in IGF-I treated animals. Without the litter, the prolactin levels dropped to an equally low basal value in both groups. Then, the plasma prolactin levels rose during suckling, but IGF-I administration significantly attenuated the elevation in plasma prolactin levels at 15, 30 and 60 min after the pups were returned (***p < 0.001; **p < 0.01, t = 4.124, t = 4.804 and t = 3.322, respectively). The results are presented as the mean values ± SEM.

TIDA neurons play a key role in the hypothalamic control of prolactin secretion. Therefore, we investigated whether maternally elevated IGFBP-3 plays a role in this regulation by sequestering IGF-I in a functionally inactive complex. IGF-I and ACSF, as a control, were administered i.c.v. to rat mothers via osmotic minipumps in the same composition and concentration as described above. The serum prolactin levels induced by suckling were measured on the 14^th^ postpartum day. Before the pups were removed for 4 h, the prolactin concentrations were 166.3 ± 30.3 and 103.96 ± 22.35 ng/ml in the ACSF- and IGF-I-treated groups, respectively. The plasma prolactin concentration was reduced to basal levels by the end of a subsequent 4-hour separation period (Fig. 5). The pups were then returned to the mother at 0 min, which led to almost immediate nursing and suckling and in turn resulted in dramatically elevated serum prolactin levels (Fig. 5). We performed a two-way repeated measures ANOVA to examine if IGF-I has an effect on suckling-induced prolactin release. The effect of treatment was significant (p < 0.001, F = 33.85), and the interaction of time and treatment as well (p < 0.01, F = 3.34). Suckling induced the prolactin release significantly in both groups (effect of time: p < 0.001, F = 17.69). We conducted Newman-Keuls post hoc test to assess the specific times, at which a difference was found between ACSF and IGF-I treated groups. At 15, 30 and 60 min after the pups were returned, the prolactin level was significantly reduced by IGF-I with the following p values: p = 0.0013, p = 0.0002, and p = 0.0167, respectively. These results indicate that prolonged administration of IGF-I can suppress the suckling-induced prolactin release in mother rats. The reduced prolactin secretion was also reflected by the reduced weight gain of the litters, which was 11.1 ± 1.4 g for the ACSF-treated and 7.5 ± 1.8 g for the IGF-I-treated mothers (p = 0.013).

### IGF-I administration induces TH expression in the arcuate nucleus of mother rats *in vivo*

**Figure 6:**
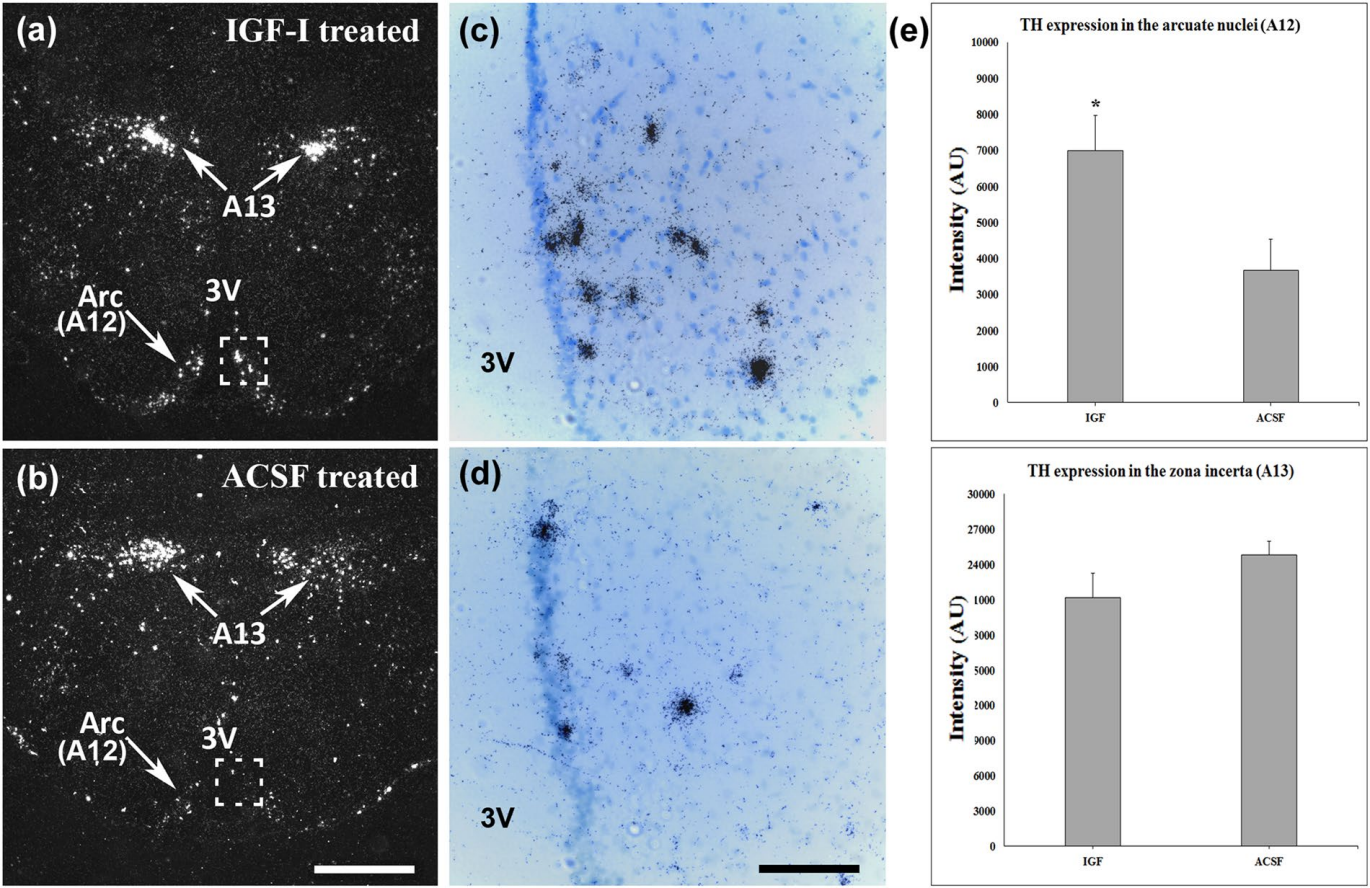
I.c.v. IGF-I administration enhanced the expression of TH in the arcuate nucleus. (**a,b**) The brains of IGF-I- and ACSF-treated rat mothers (n = 4 in both groups) were used for *in situ* hybridization histochemistry. Dark-field photomicrographs show that TH mRNA expression (white dots) was abundant in the zona incerta (A13 dopamine cell group) and the arcuate nucleus (A12 dopamine cell group) in IGF-I treated mothers. Without IGF-I, however, TH mRNA levels are low in the arcuate nucleus and are abundant in the zona incerta. (**c,d**) High magnification bright-field photomicrographs show the corresponding framed areas – the dorsomedial subdivision of the arcuate nucleus – in A and B to demonstrate increased density of individual autoradiography grains (black dots) in IGF-I-treated mothers. (**e**) Quantification of the signal in TH *in situ* hybridization histochemistry. The intensity in the arcuate nuclei of IGF-I treated mothers exhibited an approximate twofold increased compared with that in the ACSF-treated group (n = 6–6, *p < 0.05, p = 0.0435), while a significant difference was not detected in the zona incerta. The results are presented as the mean values ± SEM. Abbreviations: Arc – arcuate nucleus, A12–A13 – dopamine cell groups in the arcuate nucleus, and the zona incerta, respectively, 3 V – third ventricle. Scale bar = 1 mm for A and B and 100 µm for C and D.

We addressed the potential mechanisms involved in the actions of IGF-I in the suppression of prolactin release. TH, which is the rate-limiting enzyme in dopamine synthesis, regulates dopamine levels in dopaminergic cells. Therefore, we used *in situ* hybridization histochemistry to determine the expression level of TH mRNA in TIDA neurons (A12 cell group), which play a crucial role in the regulation of pituitary prolactin secretion, and in dopaminergic cells in the zona incerta (A13 cell group), which do not participate in the control of prolactin secretion. TH expression was quantified using densitometry in the brains of IGF-I- and ACSF-treated rat dams. A twofold elevation in TH mRNA expression was found in IGF-I-treated mothers in the arcuate nucleus compared with that in the control group (Fig. 6). By contrast, there was no significant difference between the two groups in the zona incerta (Fig. 6).

### IGF-I treatment enhances TH expression and increases site-specific phosphorylation *in vitro*

**Figure 7:**
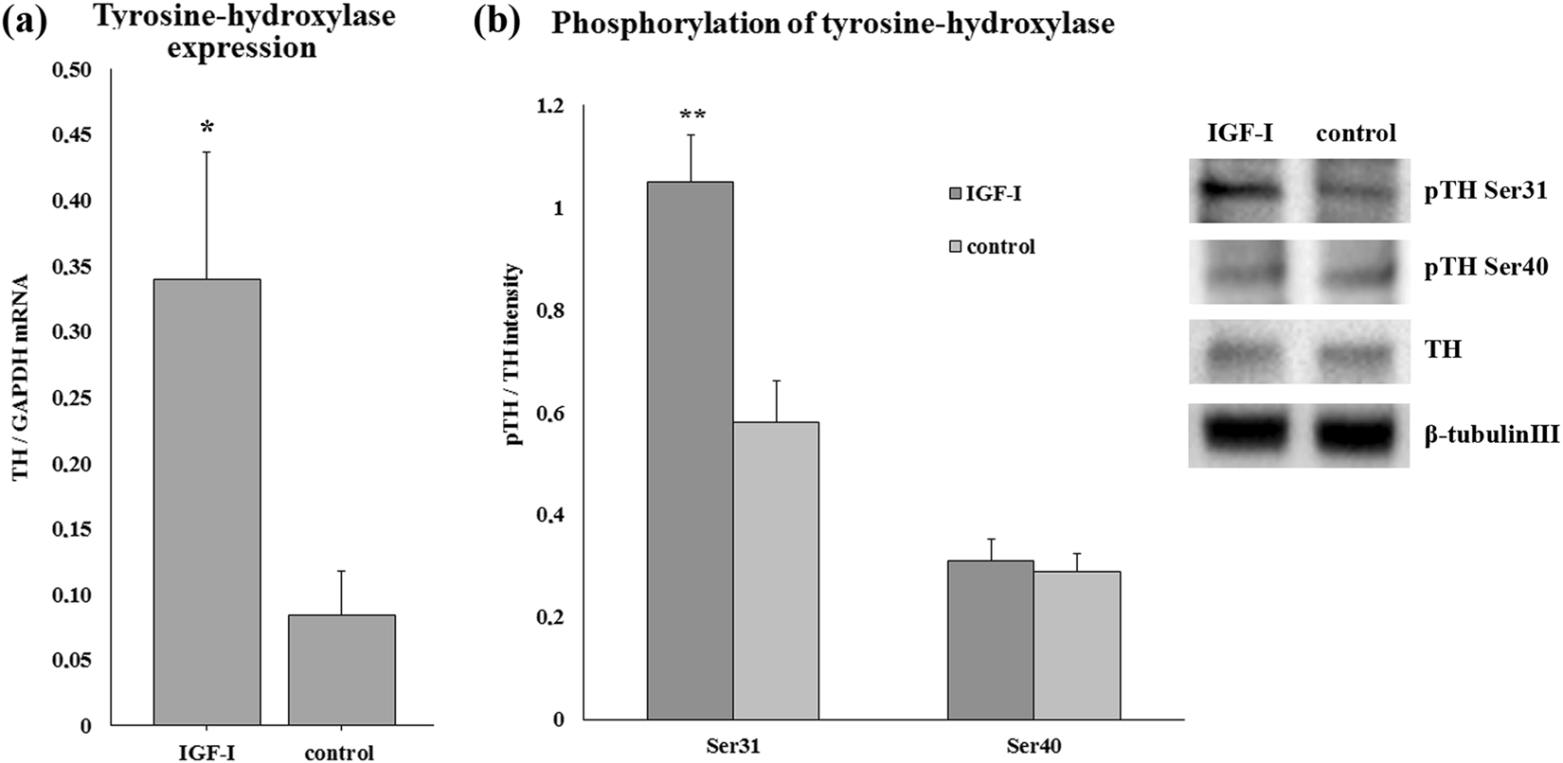
IGF-I treatment enhances the expression of TH and its phosphorylation on phosphorylation site Ser 31 in primary mediobasal hypothalamic cell cultures. (**a**) The TH mRNA expression was approximately fourfold higher in cell cultures treated with IGF-I (12.5 µg/ml) for 4 days compared with that in controls (p = 0.048). Data are expressed as the ratio of GAPDH mRNA levels (n = 4 in both groups). (**b**) Following incubation with or without IGF-I (12.5 µg/ml) for 30 min, the cells were lysed, and the phosphorylation of TH at the Ser 31 and 40 residues was determined using phosphorylation site-specific antibodies. Data are expressed as the ratio of TH intensity. The panels beside the diagram show representative images of immunoblot bands. Western blot results show that IGF-I treatment significantly increased the phosphorylation of TH at the Ser 31 residue (p = 0.003). There was no difference between the two cultures at the Ser 40 phosphorylation site (n = 6 for both groups). The results are presented as the mean values ± SEM.

Mediobasal hypothalamic primary cell cultures were prepared from newborn rats and incubated for 4 days with IGF-I-containing (12.5 µg/ml) or control medium. IGF-I administration significantly (p = 0.048) enhanced the TH mRNA expression – measured by real time RT-PCR – compared with that in the control cultures, which were treated with control medium (Fig. 7a).

Another group of hypothalamic cell cultures was grown in control medium for 4 days, and on the 5^th^ day, half of them were incubated in IGF-I-containing medium for 30 min at the same concentration as described above. Using Western blotting with phosphorylation-site specific antibodies, we determined that IGF-I stimulated a significant increase in the phosphorylation of the Ser 31 residue when compared with that in cells incubated under basal conditions. In contrast, IGF-I did not enhance the phosphorylation of the Ser 40 residue (Fig. 7b).

## Discussion

We have now evaluated our previous microarray study, which was focused on a single gene^16^, and identified 5 additional genes with highly significant and over 2-fold increases in mother rats. The results were compared with those of a similar microarray study^18^ in which 27 maternally induced genes involved in CNS development were identified in mice. There are some differences between the designs of the two studies, including rats vs. mice, postpartum day 9 vs. 7, non-maternal females vs. virgin females as the control group, and dissected tissue from the whole preoptic area vs. only the medial preoptic area. Despite these differences, we identified a gene, insulin-like growth factor binding protein-3 (IGFBP-3), which was elevated in mother rats in both studies. Because of the high rate of false positive data in microarray experiments^34^, we measured the mRNA level of IGFBP-3 in rat dams and pup-deprived controls with 2 independent methods, RT-PCR and *in situ* hybridization histochemistry, as IGFBP-3 induction has not been validated in the previous studies. Both methods validated the increased IGFBP-3 expression level in the preoptic area in lactating rat dams. The confirmed maternal elevation in IGFBP-3 expression and its distribution in the region of the preoptic area involved in maternal behaviour, the medial preoptic area^35^, suggest its involvement in the control of maternal behaviour. There are several different neuronal populations in this area that contribute to this regulation network, including galanin-, amylin- or melanin-concentrating hormone (MCH)-expressing neurons^8, 17, 36^. Of these populations, the distribution of IGFBP-3 in the MPOA is similar to the expression pattern of MCH. The expression of MCH increases gradually until the weaning of pups^36, 37^ and has been suggested to be involved in the termination of maternal responsiveness during the late postpartum period^38^.

Apart from the preoptic area, IGFBP-3 expression was also detected in other hypothalamic sites by *in situ* hybridization histochemistry, suggesting additional functions. The role of IGFBP-3 in the PVN is unlikely to be related to maternal modifications as the expression level did not change in mothers. This finding also suggests that the induction of IGFBP-3 may be specific to certain cell types. Indeed, IGFBP-3 was induced in the dorsomedial subdivision of the arcuate nucleus, specifically in TH-positive dopaminergic cells. These cells are known to regulate prolactin release from the pituitary^39^, which suggests an additional role for IGFBP-3 in lactation. The IGFBP-3 distribution data in the hypothalamus have not been reported before. A previous study focusing on the effect of hypoxia on IGFBP-3 reported its expression in the brain but described only cortical and thalamic locations^40^.

The distribution of IGFBP-3 suggests that it may participate in the control of maternal behaviours and lactation. IGFBP-3 binds IGF-I with high affinity, thereby reducing the level of bioavailable IGF-I. To examine the role of IGFBP-3 in the regulation of maternal behaviours and lactation, we inhibited this IGF-sequestering effect with continuous and prolonged i.c.v. infusion of either IGF-I or an IGFBP ligand-binding inhibitor, NBI-31772^41^. These infusions presumably increased the hypothalamic bioavailability of IGF-I. Although peripherally administered IGF-I reduces the brain level of IGF-I^42^, we used continuous central infusions to ensure its permanently high bioavailability in the hypothalamus. To administer the drugs, we used osmotic minipumps with a continuous 0.5 μl/hour flow rate for a maximum of 14 days. Another reason why we preferred prolonged IGFBP-3 inhibition is that slow, neuroplastic effects can also be detected. For example, acute IGF-I and NBI-31772 administration have anxiolytic and antidepressant-like effects^41, 43^, while their prolonged administration can affect neuronal plasticity and neural survival in dopaminergic neurons^44, 45^. We did not detect the anxiolytic effects of these agents following their long-term presence as neither affected the number of open arm entries in the elevated plus-maze test, a marker of anxiety-like behaviour.

The effect of IGFBP-3 inhibition in the brain on maternal behaviour was examined. The mothers showed several aspects of normal behaviour, demonstrating that they can perform maternal behaviours. However, we measured an increased latency to carry the first pup and the last pup into the nest following the sequestration of IGFBP-3. Since moving the pups into the nest is the most frequently used indicator of maternal motivation as a goal-directed behaviour^35^, our results suggest that elevated expression of IGFBP-3 in the MPOA in lactating dams contributes to maternal motivation. Different factors are known to contribute to the initiation and maintenance of maternal behaviour. While peripartum hormonal changes initiate and potentiate the onset of maternal motivation, they are not required for maintenance of maternal care. Continuous exposure to pups and afferent stimuli from them are sufficient for supporting the maintenance of maternal motivation during the postpartum period^15^. The neurochemical regulatory mechanisms, which contribute to ongoing maternal care, are much less established. Based on our results, IGF-I and IGFBP-3 appear to contribute to the maintenance of maternal behaviours, thereby suggesting a previously unknown mechanism involved in maternal regulations. The lack of effects of prolonged IGF-I and NBI-31772 on activity in the elevated plus-maze test indicates that reduced maternal motivation was not a consequence of altered state of anxiety, which itself could affect maternal behaviour.

Based on the induction of IGFBP-3 in TH neurons of the arcuate nucleus, we also addressed its role in lactation. In rodent mothers, lactation is driven by the suckling pup stimuli, which evokes a dramatic increase in serum prolactin levels^46^. We observed a high degree of elevation in control mothers, which received ACSF injection. However, prolactin levels increased to a significantly lesser degree after the sequestration of IGFBP-3, supporting a role of IGFBP-3 in the regulation of prolactin secretion. Since prolactin increased significantly even in the presence of IGF-I in response to suckling, it is not possible to determine if a higher concentration of IGF-I could have blocked it completely or if prolactin has an IGF-I-independent component. At the same time, as prolactin levels were reduced in the presence of IGF-I, the body weight of pups gained during 1 hour of suckling was also decreased in the IGF-I group compared with that in the controls. These are new findings as hypothalamic IGF-I and IGFBP-3 have not been shown to control prolactin secretion in mothers. Previous studies have revealed that IGF-I acts on lactation at the level of the pituitary by supporting the differentiation and proliferation of lactotroph cells^47, 48^. The involvement of hypothalamic IGF-I in reproductive neuroendocrine functions has been suggested before; it plays a role in the regulation of gonadotropin-releasing hormone (GnRH) cell function. Infusion of IGF-1 stimulated secretion of GnRH^32, 49^, while its antagonism impaired oestradiol-positive feedback and luteinizing hormone (LH) surges^31, 33^.

Binding of IGF-I to its receptor results in autophosphorylation of the receptor, which in turn initiates a cascade of cellular signal transduction events. One key step is the binding of insulin receptor substrate (IRS)-1 to phosphotyrosine residues on the receptor. IRS-1 then acts as a docking protein for the downstream signal transduction components, including the Ras/ERK1/2 and PI3K/Akt pathways. IGF-I supports the survival of dopaminergic neurons through these two pathways, which has mostly been investigated in the nigrostriatal dopamine system^45, 50–52^. IGF-I gene therapy in female rats reversed their hypothalamic DA dysfunction and hyperprolactinemia^53^. In line with these data, IGF-I enhanced TH expression in our hypothalamic cell culture experiments and in lactating rat dams. In dopaminergic neurons, activated ERK1/2 proteins enhance the transcriptional activation of TH by nuclear receptor related-1 (Nurr1)^54, 55^, which therefore represents a possible mechanism of the effect of IGF-I on TIDA neurons in our study. In addition, we also found an increase in the level of phosphorylation of the TH enzyme at the Ser 31 phosphorylation site by IGF-I *in vitro*. This finding suggests rapid enhancement of dopamine synthesis by IGF-I as phosphorylation of TH results in an increased catecholamine production^56^. This rapid action of IGF-I on dopaminergic neurons has not been previously described. Rat TH can be phosphorylated at the four following different sites: Ser 8, 19, 31, 40, among which IGF-I action is selective for site 31 over 40. ERK1/2 serine kinases are mainly responsible for Ser 31 phosphorylation^57^, suggesting that IGF-I may have acted via ERK1/2 serine kinases to induce phosphorylation of TH.

To exert these actions, IGF-I is present in the hypothalamic extracellular space at a relatively high concentration^58^. IGF-I in this region could originate in neurons, which express IGF-I in the hypothalamus^59^, and in the circulation as IGF-I can reach the hypothalamus by crossing the blood-brain-barrier or blood-cerebrospinal fluid barrier^60^. IGF-I in the plasma shows decreased levels during pregnancy because of reduced growth hormone (GH) receptor expression in the liver. After delivery, circulating IGF-I reaches its original, higher levels^61^. The suckling stimulus is known to cause a rapid and transient increase in plasma GH^62^ but does not result in any significant difference between lactating and pup-deprived animals^61^. These data suggest that the IGF-I concentration in the hypothalamus does not differ between lactating, pup-deprived mothers and virgin females, so its effects must be modified by other mechanisms that alter its concentration.

**Figure 8:**
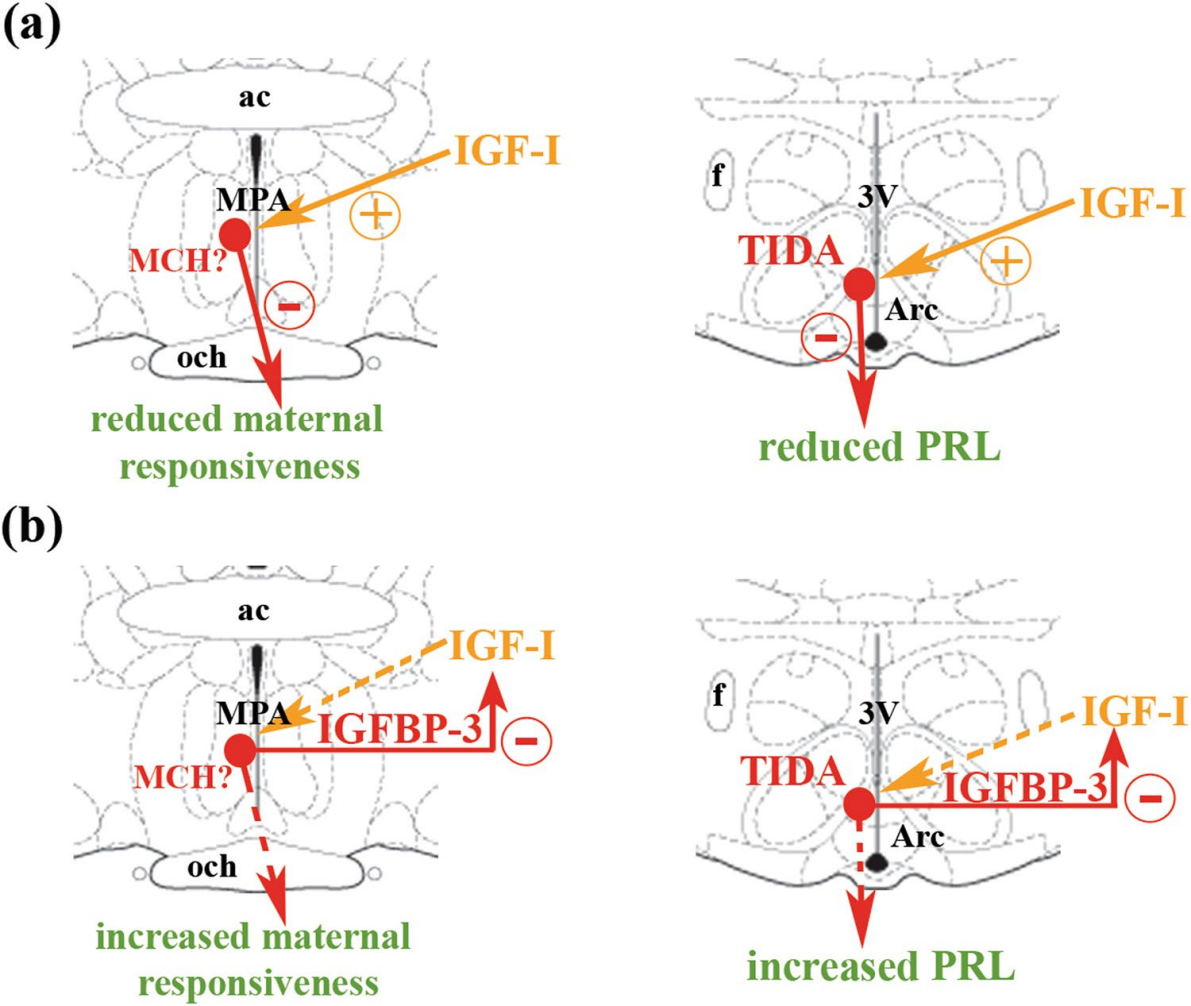
A model showing how the IGF-I - IGFBP-3 system regulates maternal responsiveness and prolactin release. (**a**) In virgin females and pup-deprived mothers, IGF-I of hypothalamic origin or from the circulation probably induce the expression of MCH in the preoptic area and enhances the expression of the TH enzyme, furthermore stimulates its activation by phosphorylation in TIDA neurons. Consequently, these cells produce more MCH or dopamine, which inhibits maternal responsiveness or prolactin secretion from the pituitary. (**b**) In lactating mothers, MCH and TIDA neurons enhance the expression of IGFBP-3, which can neutralize IGF-I. The MCH or TIDA stimulating effect induced by IGF-I diminishes, which results in a decrease in MCH or dopamine production and concomitant increased maternal responsiveness or prolactin secretion due to its release from tonic inhibition. Abbreviations: ac – anterior commissure, Arc – arcuate nucleus, f – fornix, MPA – medial preoptic area, och – optic chiasm, 3 V – third ventricle.

The elevated expression of IGFBP-3 could play an important role in decreasing effective IGF-I levels in the brain extracellular space by binding to IGF-I and neutralizing it. This mechanism has been suggested to underlie the effect of IGF-I on tau phosphorylation in Alzheimer’s disease; amyloid-β stimulates local astrocytes to release IGFBP-3, which in turn inhibits IGF-I-mediated suppression of tau phosphorylation^63^. We propose a similar mechanism in mother rats based on the existence of intrinsic IGF-I and IGFBP-3, which compose a system to regulate maternal changes in the brain (Fig. 8). In this model, IGF-1 inhibits maternal responses, and during the peri- and postpartum periods, the elevated expression of IGFBP-3 leads to the elimination of the inhibitory effects of IGF-I (Fig. 8). Since IGFBP-3 is expressed in TIDA neurons and probably in MCH neurons of the preoptic area, IGFBP-3 can be released specifically from these cells in lactating dams to prevent the stimulatory action of IGF-I on these cells.

The regulation of the actions of a hypothalamic hormone by its binding proteins has also been previously suggested in other systems. For example, the corticosteroid-binding globulin expressed in the brain can direct the action of the stress hormone^64, 65^. The central distribution of sexual hormone-binding globulin (SHBG) is also known^66^. In the hypothalamus, SHBG expression shows alterations according to the reproductive state, so it may play role in modulating the central actions of ovarian steroids^67^. However, we also established state-dependent induction of IGFBP-3 to regulate the actions of IGF-I actions on these steroids. This hypothesis represents a novel mechanism in neuroendocrinology; neurons can block the effects of a hormone by inducing the expression of its binding protein. Nevertheless, the recently suggested role of IGF-independent effects of IGFBP-3^21–23^ in maternal brain alterations cannot be excluded.

*In conclusion*, the major finding of this study is that prolonged IGF-I treatment reduced suckling-induced prolactin release and maternal responsiveness. In turn, IGFBP-3 expression shows marked elevation in well-defined regions of the maternal brain, including the MPOA and the arcuate nucleus. The role of this elevated IGFBP-3 may be the neutralization of the effect of IGF-I on distinct neurons by binding and sequestering it. Preventing the binding of IGFBP-3 to IGF-I with the ligand inhibitor NBI-31772 significantly suppressed maternal motivation. In addition, IGF-I also inhibited suckling-induced prolactin release. IGF-I can exert this action by increasing the dopamine release of TIDA neurons as IGF-I stimulated TH expression and phosphorylation. IGFBP-3 can reduce this action of IGF-I as TIDA neurons in the arcuate nucleus expressed a markedly elevated level of IGFBP-3 in lactating mothers. Thus, IGFBP-3 functions as an intrinsic regulator of lactation by switching TIDA neurons to a mode that permits induction of PRL release from the pituitary. Therefore, the IGF-I - IGFBP-3 systems can play an important role in the different aspects of the adaptation of the female brain to motherhood by a novel neuroendocrine mechanism.

## Methods

### Animals

All animal experimentations were approved by the Animal Examination Ethical Council of the Animal Protection Advisory Board at the Semmelweis University, Budapest, and met the guidelines of the Animal Hygiene and Food Control Department, Ministry of Agriculture, Hungary. A total of 85 female Wistar rats (250–300 g adult body weight; Charles Rivers Laboratories, Budapest, Hungary) were used in this study (Table 3). Animals were kept on standard laboratory conditions with 12-h light, 12-h dark periods (lights on at 06:30), and supplied with dry rat food and drinking water ad libitum. Rats were housed three per cage at a temperature of 22 ± 1 °C before experiments. For mating, two female and a male rat were kept in a cage for 7 days. After that, potentially pregnant female rats as well as dams with litter and their pup-deprived counterparts were kept in cages individually. Rats were anesthetized between 9 and 10 AM with an intramuscular injection of anaesthetic mix containing 0.3 mL/300 g body weight ketamine (67 mg/kg) and 0.2 mL/300 g body weight xylazine (13 mg/kg) before implantation of osmotic minipumps or jugular cannulae, decapitation and cardiac perfusion.

**Table 3:** Summary and timing of the experiments with specification of the number of used animals in each experiment.

Objective	Number of rats	Postpartum day	Experimental procedure
Measurement of IGFBP-3 mRNA level	9 lactating mothers *vs*. 8 pup-deprived mothers	11^th^ day	RT-PCR
Establishment of the expression pattern and level of IGFBP-3	6 lactating mothers *vs*. 6 pup-deprived mothers	11^th^ day	*In situ* hybridization histochemistry (ISHH)
Examination of the expression of IGFBP-3 in dopaminergic neurons	5 lactating mothers	11^th^ day	Combination of ISHH for IGFBP-3 and immunohistochemistry for TH
Determination of the role of IGF-I system in maternal behaviour and TH expression level	10 IGF-I-treated *vs*. 10 ACSF-treated, and 8 NBI-31772-treated *vs*. 8 DMSO-ACSF-treated (control) lactating mothers	2^nd^ day	Implantation of osmotic minipumps
		6^th^ day	Pup-retrieval test
		7^th^ day	Elevated plus-maze test
		4–9^th^ days	Examination of undisturbed maternal behaviour
	4 IGF-I-treated *vs*. 4 ACSF-treated lactating mothers	14^th^ day	*In situ* hybridization histochemistry
Determination of the role of IGF-I system in prolactin release	7 IGF-I-treated *vs*. 8 ACSF-treated (control) lactating mothers	2^nd^ day	Implantation of osmotic minipumps
		13^th^ day	Implantation of jugular cannulae
		14^th^ day	Blood sampling for prolactin measurement

### Microdissection of brain tissue samples

On the 11^th^ day postpartum, brains were dissected from 9 primiparous lactating and 8 pup-deprived rat dams. Thick coronal brain sections were prepared around the preoptic area with a razor blade cut immediately rostral to the optic chiasm and 2 mm caudal to this level (Fig. 2a). A horizontal cut immediately above the anterior commissure, and sagittal cuts on both sides of the brain 2  mm lateral to the midline were used to dissect tissue. Dissected area contained the preoptic area of the hypothalamus as well as small parts of adjacent brain structures including parts of the diagonal band of Broca, the anterior commissure, the optic tract, the ventral pallidum. In this study, however, we refer to this tissue block as the preoptic area. The dissected tissue samples were quickly frozen on dry ice, and stored at −80 °C.

### Real-time RT-PCR

Real-time RT-PCR was carried out as described previously^16^. Briefly, Total RNA was isolated from frozen preoptic tissue samples, or lysated primer cultures. The concentration of RNA was adjusted to 2 µg/µL, and it was treated with Amplification Grade DNase I (Invitrogen). Then, cDNA was synthesized using SupersciptII (Invitrogen) as suggested in the kit protocol. The cDNA was subsequently diluted (10x), and 2.5 µL of the resulting cDNA was used as template in PCR reactions using SYBR Green dye (Sigma, St Louis, MO, USA). The PCR reactions were performed with iTaq DNA polymerase (Bio-Rad Laboratories, Hercules, CA, USA) and GAPDH was used as housekeeping gene. The primers were: ACAGCCAGCGCTACAAAGTT and GCGGTATCTACTGGCTCTGC for IGFBP-3, and GCTACCGAGAGGACAGCATC and GCACCATAAGCCTTCAGCTC for TH, and TGCCACTCAGAAGACTGTGG and GTCCTCAGTGTAGCCCAGGA for GAPDH. Cycle threshold (Ct) values were obtained from the linear region of baseline adjusted amplification curves. The GAPDH related values were calculated using the following formula: log(Ct_(GAPDH)_ − Ct_(IGFBP3 or TH)_). Statistical analyses were performed by unpaired t-test for comparisons of the two different groups.

### Production of *in situ* hybridization probe for IGFBP-3 and TH

Preparation of the *in situ* hybridization probes was performed as described previously^68^. Briefly, PCR products of amplification of IGFBP-3 and TH were produced from hypothalamic cDNA using the following primer pairs for IGFBP-3: A: ACAGCCAGCGCTACAAAGTT and GCGGTATCTACTGGCTCTGC, B: CCTTGTTGGAGACCCTGGTA and TCACACCCTGTATTGCCAGA; and the following for TH: GCTACCGAGAGGACAGCATC and GCACCATAAGCCTTCAGCTC. The PCR products were purified from gel, inserted into TOPO TA cloning vectors (Life Technologies) and transformed chemically into competent bacteria. Selected plasmids were applied as templates in PCR reactions, using the primer pairs specific for IGFBP-3 and TH, respectively, with the reverse primers also containing a T7 RNA polymerase recognition site. At the end, the identities of the cDNA probes were verified by sequencing.

### *In situ* hybridization histochemistry

To describe the expression pattern of IGFBP-3 and also to measure the expression level of IGFBP-3, brains of 6 primiparous lactating mothers and 6 age-matched pup-deprived mothers were removed at 11 days postpartum. In addition, brains of 4 IGF-I and 4 ACSF-treated rat mothers were removed at the end of the treatment, at 14 days postpartum to examine the effects of prolonged i.c.v. IGF-I administration on TH expression levels. The fresh tissue was quickly frozen on dry ice. *In situ* hybridization histochemistry was processed as described previously^16^. Briefly, serial coronal sections (12 µm) were cut using a cryostat from bregma level + 3.5 mm to −6 mm, mounted on positively charged slides (SuperfrostUltraPlus™; Thermo Fisher Scientific, Pittsburgh, PA, USA), dried, and stored at −80 °C until use. Antisense [35 S]UTP-labelled riboprobes were generated using T7 RNA polymerase of the MAXIscript *In vitro* transcription kit (Ambion, Austin, TX) from PCR-amplified fragments of the cDNA subcloned into TOPO TA vectors. Tissue was prepared using an mRNA-locator Kit (Ambion) according to manufacturer’s instructions. For hybridization, we used 80 µl hybridization buffer and 1 million DPM of labelled probe per slide. Washing procedures included a 30 min incubation in RNase A, followed by decreasing concentrations of sodium-citrate buffer (pH = 7.4) at room temperature, and then at 65 °C. After drying, slides were dipped in NTB nuclear track emulsion (Eastman Kodak, Rochester, NY), stored for 3 weeks at 4 °C for autoradiography. Then, the slides were developed and fixed with Kodak Dektol developer and Kodak fixer, respectively, counterstained with Giemsa, dehydrated, and coverslipped.

### Densitometric analysis of *in situ* hybridization histochemistry signal

Dark-field photomicrographs were taken of the sections where IGFBP-3 and TH signal was the highest in the medial preoptic area, and arcuate nucleus and zona incerta, respectively, using a 10x objective. Each image was divided into 2 halves with identical size, such that one half contained the observed autoradiography signals, while the other half served as background control. The pixel number of these areas was calculated using ImageJ 1.47 v (National Institutes of Health, USA) software. The difference between the 2 values (the half picture containing labeled cells - the half picture containing only background autoradiography signal) was used to quantify IGFBP-3 and TH mRNA levels. mRNA levels in the 2 groups were compared using two-tailed unpaired t-test.

### Implantation of intracerebroventricular cannulae

On the 2^nd^ day postpartum, rats were divided into 4 groups to determine the role of IGF-I system in maternal behaviour and TH expression level: icv. infusion of ACSF (n = 10), IGF-I dissolved in ACSF (n = 10), 1% DMSO in ACSF (n = 8), NBI-31772 (n = 8) dissolved in ACSF containing 1% Dimethyl sulfoxide (DMSO). In another experiment, 7 IGF-I-treated *vs*. 8 ACSF-treated (control) mothers were used to determine the role of IGF-I system in prolactin release. Osmotic minipumps injecting continuously for 14 days (ALZET Micro-Osmotic Pump Model 2002, Durect™) were used loaded with IGF-I (4 µg/µl; 48 µg IGF-I/day, PeptideSciences™), NBI-31772 (1.66 µg/µl; 19,92 µg NBI-31772/day, Merck™), ACSF (147 mM NaCl, 3.5 mM KCl, 2 mM CaCl2, 1 mM MgCl2, pH = 7.2), or 1%DMSO in ACSF connected to cannulae according to manufacturer’s guide. For intracerebroventricular (i.c.v.) implantation of the cannulae, rats were anesthetized with 0.2 ml xylazine and 0.3 ml ketamine and fixed in a stereotaxic apparatus. The skin was cut over the skull and a hole of about 1 mm diameter was drilled into the left side of the skull above the lateral ventricle positioned at the following coordinates: antero-posterior, −0.5; lateral, 1.4; ventral, 3.6 mm. Cannulae (ALZET Brain Infusion Kit 2, Durect™) were inserted into the lateral ventricle and fixed to the skull with cranioplastic cement and the pumps placed subcutaneously (s.c.) at the back of the animal. After the operation, Tardomyocel® comp. III antibiotics (0.1 ml/kg body weight) was given s.c. to the animals for 5 days to prevent infections.

### Undisturbed maternal behaviour

We used 36 mother rats injected i.c.v. with osmotic minipumps to investigate their undisturbed maternal behaviour. The behaviour of each dam was recorded as described previously^69^. Briefly, the undisturbed maternal behaviour was observed for three 60 min daily observation periods, 5 days long, starting on 4^th^ day postpartum, after one day recovery following the implantation of osmotic minipumps. Observations were performed at two periods during the light phase (8:00 and 13:00 h, lights ON at 06:30) and at one period during the dark phase (19:30 h, lights OFF at 18:30). Within each observation period, the behaviour of each mother was scored 20 times spaced 3 min each one (20 observations × 3 periods per day × 5 days = 300 observations/mother) as present or absent. The following behaviours were scored as present or absent: high kyphosis (HK): mother nursing pups in an arched-back posture with rigid limbs; licking/grooming (LG): mother licking–grooming any pup (body + anogenital region); prone nursing (PN): mother nursing in a “blanket” posture in which the mother just lies over the pups, but did not have her back arched and there was no obvious extension of her legs; supine nursing (SN): mother nursing in a “passive” posture in which the mother lies on her back or side while the pups are nursed; out of the nest (0): mother out of the nest (no maternal contact). Number of each behaviour/300 observation ratio was calculated for every mother. Normality was tested with Kolmogorov-Smirnov test while difference of the mean was analyzed with unpaired two-tailed t-test.

### Pup retrieval test

We used 36 mother rats injected i.c.v. with osmotic minipumps for testing. On postnatal day 6, all pups were separated from their mothers for 10 min. Subsequently, 3 pups were returned to the mother’s cage in 3 different corners of the cage away from the nest. The mother was visually observed for 5 min. The time required for the mother to carry the first and the third pup to the nest was recorded. Normality was tested with Kolmogorov-Smirnov test. Results were analyzed with two-tailed Mann-Whitney-, or unpaired t-test with Welch correction.

### Elevated plus-maze test (EPM)

To measure anxiety, dams were exposed to EPM on postnatal day 7 during the afternoon. The EPM was made of plastic, painted black and elevated 80 cm above the floor (arm length 50 cm; arm width 15 cm; central platform 15 × 15 cm; closed arm walls height 70 cm). Surface of maze was washed with water, alcohol and dried before the next animal was introduced. The duration of the test was 5 min. Rats were introduced in the centre of the maze facing a closed arm. Percentage of time spent in open arms and percentage of open arm entries (number of open arm entries/number of open plus closed arm entries) were calculated and used as measures of anxiety (entry: at least two third of the body in an arm). Normality was tested with Kolmogorov-Smirnov test while difference between the means was analyzed with two-tailed unpaired t-test.

### Combination of *in situ* hybridization histochemistry and immunohistochemistry

Brains of 5 lactating mother rats perfusion fixed on postpartum day 11 were used to examine the expression of IGFBP-3 in dopaminergic neurons. *In situ* hybridization histochemistry was performed as described above except that 20 mm thick sections were used. Following hybridization with IGFBP-3 probe and before dipping the sections in nuclear track emulsion, TH immunohistochemistry was carried out in humid chambers. Slides were first treated with mouse anti-TH antibody **(**dilution 1:1000; Chemicon, MAB5280), then in horse anti-mouse IgG (1:1000; Vector Laboratories™, Burlingame, CA, USA) for 1 h, then in ABC complex (1:500; Vector Laboratories™,) for 2 h. Finally, the sections were visualized with 3,3-diaminobenzidine (DAB) reaction.

### Microscopy and image processing

Sections were examined using an Olympus BX60 light microscope equipped with a dark-field condenser. Images were captured at 2048 × 2048 pixel resolution with a SPOT Xplorer digital CCD camera (Diagnostic Instruments, Sterling Heights, MI) using 4–40 X objectives. Images were adjusted using the “levels” and “sharpness” commands in Adobe Photoshop CS 8.0. Full resolution of the images was maintained until the final versions, which were adjusted to a resolution of 300 dpi.

### Implantation of jugular cannulae

On the 13^th^ postpartum day - under ketamine-xylazine anaesthesia (described above) – 15 dams (7 IGF-treated, 8 ACSF-treated) received 25-mm-long sterile polyethylene jugular cannulae (Plastics One). A ventral cervical skin incision was made right of the midline with its caudal terminus at the level of the clavicle. The right common jugular vein was mobilized, and cannulae were inserted into the vessel and secured in place with suture. Incisions were made on the skin at the midline between the scapulae, and jugular cannulae were pulled through the scapular incisions. The cannulae were filled with heparinized saline and sealed with metal pins.

### Blood sampling before and during suckling

On the next day we obtained blood via jugular cannulae to measure serum prolactin levels induced by suckling: first before taking away the pups for 4 h, second before returning the pups (baseline control) and also 5, 15, 30, 60 min after the return. The volume of blood taken was 0.3–0.3 ml each time, and the same amount of heparinized saline was injected back into the circulation. Blood was centrifuged at 4 °C for 10 min at 12.000 g and the supernatant was stored at −20 °C for measurement of prolactin plasma concentrations by radioimmunoassay.

### Prolactin assay

Prolactin assay was performed as described previously^70^. Briefly, the chloramine-T method was used for iodination, and protein A (BactASorb, Human Rt, Gödöllő, Hungary) was used to separate bound and free hormone. LKB Clinigamma software was used for data collection and calculations for curve fitting. Within-assay variance was 10%. Between-assay variance was 14%. The sensitivity of the prolactin assay was 0.5 ng/ml rat plasma (or 25 pg prolactin). All samples were analyzed in duplicate using 50 ml of plasma for each measurement.

### Statistical analysis of the prolactin assay

Statistical analyses were performed using Prism 5 for Windows (GraphPad Software, Inc., La Jolla, CA). Normality was tested with Kolmogorov-Smirnov test. For the suckling experiment, plasma prolactin levels of the 2 groups were compared using repeated measures two-way ANOVA to evaluate whether suckling and prolonged administration of IGF-I had an effect on the prolactin level. For posthoc comparisons, Newman-Keuls test were used.

### Preparation of hypothalamic cell cultures

10 pathogen-free newborn Wistar rats (Charles-River Laboratories) were decapitated right after birth and the mediobasal hypothalamus was excised. The tissue blocks were immediately transferred to an ice cold medium containing 2% B27, 49% Dulbecco’s modified Eagle’s medium, 49% F12 medium and penicillin-streptomycin (DMEM-F12-B27-P/S; Gibco®, Life Technologies™). First, tissue was homogenized and centrifuged for 4 min on 3000 rpm. Second, the supernatant was removed and 2 ml 0.1% Trypsin (diluted in DMEM) added to the pellet. Third, after 4 min incubation in a 37 °C water bath with Trypsin, 2 ml 0.5% DNase and 1% Bovine Serum Albumin (diluted in DMEM-P/S) was added to the digested tissue, mixed and centrifuged for 4 min at 3000 rpm. After this, supernatant was removed again, and 2 ml DMEM-F12-B27-P/S was put to the pellet, mixed and centrifuged for 4 min at 3000 rpm. This step was repeated twice and the resultant cell pellet was dispersed in DMEM-F12-B27-P/S at a density of approximately 3.9 × 10^5^ cells/ml and plated out at 0.5 ml/well onto 8 wells of a polylysine coated 24-well culture plate, which were then transferred to a 5% CO2 humidified incubator at 37 °C. After the first 24 h in culture, half of the medium (250 µl/well) was removed and changed to 25 µg/ml IGF-I (PeptideSciences™, diluted in DMEM-F12-B27-P/S) in 4 wells and DMEM-F12-B27-P/S in other 4 wells, as controls. Half of the medium was replaced every day as described above and cells were used for examination on the 5^th^ day.

### Western blot analysis

After removal of the medium, cells were washed twice with sterile PB solution and then mixed with RIPA (1 M Na-orthovanadate, 1 M NaF, Triton X100, TNE buffer: Trisma base, EDTA, NaCl, pH = 7.4) + Protease Inhibitor (RIPA + ; 40 µl PI/1 ml RIPA) using a pipette tip. Cells were collected in RIPA + and centrifuged on 4 °C at 16000 rpm. Protein concentration was measured and equilibrated to 1 µg/µL. After that Laemmli sample buffer (1% bromophenol blue, 1.5 M Trisma base, 99.5% glicerol, sodium dodecyl sulphate salt, β-mercaphtoethanol) was added to the removed supernatant and heated at 90 °C for 5 min. After running the gels and transferring the proteins to membranes (0.45 µm polyvinylidene difluoride, Amersham™ Hybond™, Life Sciences), the membranes were treated with 3% BSA solution for 2 h. Then, primary antibodies were added to membranes for 24 h (1:1000; rabbit anti-phospho TH at Ser 31, SAB4300674, Sigma; 1:10000; rabbit anti-phospho TH at Ser 40, T9573, Sigma; 1:1000, rabbit anti-TH, AB152, Millipore; and 1:2000, mouse β-tubulin-III, T8578, Sigma) and secondary for 1 h (1:10000, peroxidase labelled goat anti-rabbit or anti-mouse IgG; Jackson Immuno Research). Membranes were treated with Clarity™ Western ECL Substrate (Bio-Rad) for chemiluminescence detection, which was performed with ChemiDoc™ MP Imaging System (Bio-Rad). Intensity of bands was calculated using ImageLab software (Bio-Rad). Ratio of phospho TH – TH intensity was calculated using the following formula: (phospho TH intensity/-tubulin-III intensity)/(TH intensity/β-tubulin-III intensity) and analysed with two-tailed unpaired t-test.

## References

[1] Björklund A, Moore RY, Nobin A, Stenevi U (1973). The organization of tubero-hypophyseal and reticulo-infundibular catecholamine neuron systems in the rat brain. Brain Res..

[2] Freeman ME, Kanyicska B, Lerant A, Nagy G (2000). Prolactin: Structure, function, and regulation of secretion. Physiol. Rev..

[3] Grattan DR (2015). The hypothalamo-prolactin axis. J. Endocrinol..

[4] González-Mariscal G, Melo AI, Jiménez P, Beyer C, Rosenblatt JS (1996). Estradiol, progesterone, and prolactin regulate maternal nest-building in rabbits. J. Neuroendocrinol..

[5] González-Mariscal G, Poindron P (2002). Parental care in mammals: Immediate internal and sensory factors of control. Hormones, Brain and Behavior.

[6] Bridges RS, Ronsheim PM (1990). Prolactin (PRL) regulation of maternal behavior in rats: Bromocriptine treatment delays and PRL promotes the rapid onset of behavior. Endocrinology.

[7] Bridges RS (1996). Endocrine communication between conceptus and mother: Placental lactogen stimulation of maternal behavior. Neuroendocrinology.

[8] Dulac C, O’Connell LA, Wu Z (2014). Neural control of maternal and paternal behaviors. Science.

[9] Renier N (2016). Mapping of Brain Activity by Automated Volume Analysis of Immediate Early Genes. Cell.

[10] Numan M (2007). Motivational systems and the neural circuitry of maternal behavior in the rat. Dev. Psychobiol..

[11] Numan M, Corodimas KP, Numan MJ, Factor EM, Piers WD (1988). Axon-Sparing Lesions of the Preoptic Region and Substantia Innominata Disrupt Maternal Behavior in Rats. Behav. Neurosci..

[12] Jacobson CD, Terkel J, Gorski RA, Sawyer CH (1980). Effects of small medial preoptic area lesions on maternal behavior: Retreiving and nest building in the rat. Brain Res..

[13] Lee A, Clancy S, Fleming AS (1999). Mother rats bar-press for pups: Effects of lesions of the mpoa and limbic sites on maternal behavior and operant responding for pup- reinforcement. Behav. Brain Res..

[14] Morgan HD, Watchus JA, Milgram NW, Fleming AS (1999). The long lasting effects of electrical simulation of the medial preoptic area and medial amygdala on maternal behavior in female rats. Behav. Brain Res..

[15] Dobolyi A, Grattan DR, Stolzenberg DS (2014). Preoptic inputs and mechanisms that regulate maternal responsiveness. J Neuroendocrinol.

[16] Dobolyi A (2009). Central amylin expression and its induction in rat dams. J. Neurochem.

[17] Szabó ÉR, Cservenák M, Dobolyi A (2012). Amylin is a novel neuropeptide with potential maternal functions in the rat. Faseb J..

[18] Driessen, T. M. *et al*. Genes showing altered expression in the medial preoptic area in the highly social maternal phenotype are related to autism and other disorders with social deficits. *BMC Neurosci*. **15** (2014). 10.1186/1471-2202-15-11 PMC3906749 24423034

[19] Russo VC, Gluckman PD, Feldman EL, Werther GA (2005). The insulin-like growth factor system and its pleiotropic functions in brain. Endocr. Rev..

[20] Modric T (2001). Phenotypic manifestations of insulin-like growth factor-binding protein-3 overexpression in transgenic mice. Endocrinology.

[21] Duan C, Xu Q (2005). Roles of insulin-like growth factor (IGF) binding proteins in regulating IGF actions. Gen. and Comp. Endocrinol.

[22] Wetterau LA, Moore MG, Lee KW, Shim ML, Cohen P (1999). Novel aspects of the insulin-like growth factor binding proteins. Mol. Genet. Metab..

[23] Kalluri HSG, Dempsey RJ (2011). IGFBP-3 inhibits the proliferation of neural progenitor cells. Neurochem. Res..

[24] Honda M (2009). IGFBP3 colocalizes with and regulates hypocretin (orexin). PloS One.

[25] Rensink AAM (2002). Expression of the cytokine leukemia inhibitory factor and pro-apoptotic insulin-like growth factor binding protein-3 in Alzheimer’s disease. Acta Neuropathol..

[26] Beilharz EJ (1998). Co-ordinated and cellular specific induction of the components of the IGF/IGFBP axis in the rat brain following hypoxic-ischemic injury. Mol. Brain Res..

[27] Dyer AH, Vahdatpour C, Sanfeliu A, Tropea D (2016). The role of Insulin-Like Growth Factor 1 (IGF-1) in brain development, maturation and neuroplasticity. Neuroscience.

[28] D’Ercole AJ, Ye P, Calikoglu AS, Gutierrez-Ospina G (1996). The role of the insulin-like growth factors in the central nervous system. Mol. Neurobiol..

[29] Czech MP (1989). Signal transmission by the insulin-like growth factors. Cell.

[30] Davila D, Piriz J, Trejo JL, Nunez A, Torres-Aleman I (2007). Insulin and insulin-like growth factor I signalling in neurons. Front. Biosci..

[31] Todd BJ, Fraley GS, Peck AC, Schwartz GJ, Etgen AM (2007). Central insulin-like growth factor 1 receptors play distinct roles in the control of reproduction, food intake, and body weight in female rats. Biol. Reprod.

[32] Hiney JK, Srivastava VK, Pine MD, Dees WL (2009). Insulin-like growth factor-I activates KiSS-1 gene expression in the brain of the prepubertal female rat. Endocrinology.

[33] Sun Y, Todd BJ, Thornton K, Etgen AM, Neal-Perry G (2011). Differential effects of hypothalamic IGF-I on gonadotropin releasing hormone neuronal activation during steroid-induced LH surges in young and middle-aged female rats. Endocrinology.

[34] Wang X, Hessner MJ, Wu Y, Pati N, Ghosh S (2003). Quantitative quality control in microarray experiments and the application in data filtering, normalization and false positive rate prediction. Bioinformatics.

[35] Bridges RS (2015). Neuroendocrine regulation of maternal behavior. Front. Neuroendocrin.

[36] Rondini TA, Donato J, Rodrigues BdC, Bittencourt JC, Elias CF (2010). Chemical identity and connections of medial preoptic area neurons expressing melanin-concentrating hormone during lactation. J. Chem. Neuroanat..

[37] Knollema S, Brown ER, Vale W, Sawchenko PE (1992). Novel hypothalamic and preoptic sites of prepro-melanin-concentrating hormone messenger-ribonucleic-acid and peptide expression in lactating rats. J. Neuroendocrinol..

[38] Benedetto L, Pereira M, Ferreira A, Torterolo P (2014). Melanin-concentrating hormone in the medial preoptic area reduces active components of maternal behavior in rats. Peptides.

[39] Grattan DR, Kokay IC (2008). Prolactin: a pleiotropic neuroendocrine hormone. J Neuroendocrinol.

[40] Lee WH, Wang GM, Yang XL, Seaman LB, Vannucci SI (1999). Perinatal hypoxia-ischemia decreased neuronal but increased cerebral vascular endothelial IGFBP3 expression. Endocrine.

[41] Malberg JE (2007). Increasing the levels of insulin-like growth factor-I by an IGF binding protein inhibitor produces anxiolytic and antidepressant-like effects. Neuropsychopharmacol.

[42] Trueba-Saiz, A. *et al*. Circulating Insulin-like Growth Factor I Regulates Its Receptor in the Brain of Male Mice. *Endocrinology*, en20161468 (2016). 10.1210/en.2016-1468 27792405

[43] Hoshaw BA (2008). Antidepressant-like behavioral effects of IGF-I produced by enhanced serotonin transmission. Eur. J. Pharmacol..

[44] Mysoet J, Dupont E, Bastide B, Canu MH (2015). Role of IGF-1 in cortical plasticity and functional deficit induced by sensorimotor restriction. Behav. Brain Res..

[45] Quesada A, Lee BY, Micevych PE (2008). PI3 kinase/Akt activation mediates estrogen and IGF-1 nigral DA neuronal neuroprotection against a unilateral rat model of Parkinson’s disease. Dev. Neurobiol..

[46] Cservenák M (2010). Tuberoinfundibular peptide of 39 residues is activated during lactation and participates in the suckling-induced prolactin release in rat. Endocrinology.

[47] Hikake T, Hayashi S, Iguchi T, Sato T (2009). The role of IGF1 on the differentiation of prolactin secreting cells in the mouse anterior pituitary. J. Endocrinol..

[48] Stefaneanu L, Powell-Braxton L, Won W, Chandrashekar V, Bartke A (1999). Somatotroph and lactotroph changes in the adenohypophyses of mice with disrupted insulin-like growth factor I gene. Endocrinology.

[49] Hiney JK, Srivastava V, Nyberg CL, Ojeda SR, Dees WL (1996). Insulin-like growth factor I of peripheral origin acts centrally to accelerate the initiation of female puberty. Endocrinology.

[50] Ayadi AE, Zigmond MJ, Smith AD (2016). IGF-1 protects dopamine neurons against oxidative stress: association with changes in phosphokinases. Exp. Brain Res..

[51] Ebert AD, Beres AJ, Barber AE, Svendsen CN (2008). Human neural progenitor cells over-expressing IGF-1 protect dopamine neurons and restore function in a rat model of Parkinson’s disease. Exp. Neurol..

[52] Beck KD (1994). Functions of brain-derived neurotrophic factor, insulin-like growth factor-I and basic fibroblast growth factor in the development and maintenance of dopaminergic neurons. Prog. in Neurobiol.

[53] Hereñú CB (2007). Restorative effect of insulin-like growth factor-I gene therapy in the hypothalamus of senile rats with dopaminergic dysfunction. Gene Ther.

[54] Iwawaki T, Kohno K, Kobayashi K (2000). Identification of a potential Nurr1 response element that activates the tyrosine hydroxylase gene promoter in cultured cells. Biochem. Biophys. Res. Commun..

[55] Jacobsen KX (2008). A Nurr1 point mutant, implicated in Parkinson’s disease, uncouples ERK1/2-dependent regulation of tyrosine hydroxylase transcription. Neurobiol. Dis..

[56] Tekin I, Roskoski R, Carkaci-Salli N, Vrana KE (2014). Complex molecular regulation of tyrosine hydroxylase. J. Neural Transm..

[57] Haycock JW, Ahn NG, Cobb MH, Krebs EG (1992). ERK1 and ERK2, two microtubule-associated protein 2 kinases, mediate the phosphorylation of tyrosine hydroxylase at serine-31 in situ. Proc. Natl. Acad. Sci. USA.

[58] Yamaguchi F (1990). Insulin-like growth factor I (IGF-I) distribution in the tissue and extracellular compartment in different regions of rat brain. Brain Res.

[59] Niblock MM (1998). Distribution and levels of insulin-like growth factor I mRNA across the life span in the Brown Norway x Fischer 344 rat brain. Brain Res.

[60] Nishijima T (2010). Neuronal activity drives localized blood-brain-barrier transport of serum insulin-like growth factor-I into the CNS. Neuron.

[61] Escalada J, Sánchez-Franco F, Velasco B, Cacicedo L (1997). Regulation of growth hormone (GH) gene expression and secretion during pregnancy and lactation in the rat: Role of insulin-like growth factor-I, somatostatin, and GH-releasing hormone. Endocrinology.

[62] Wehrenberg WB, Gaillard RC (1989). Neuroendocrine mechanisms regulating growth hormone and prolactin secretion during lactation. Endocrinology.

[63] Watanabe, K. *et al*. The participation of insulin-like growth factor-binding protein 3 released by astrocytes in the pathology of Alzheimer’s disease. *Mol. Brain* **8** (2015). 10.1186/s13041-015-0174-2 PMC4670528 26637371

[64] Jirikowski GF, Pusch L, Möpert B, Herbert Z, Caldwell JD (2007). Expression of corticosteroid binding globulin in the rat central nervous system. J. Chem. Neuroanat..

[65] Sivukhina EV, Jirikowski GF (2014). Adrenal steroids in the brain: Role of the intrinsic expression of corticosteroid-binding globulin (CBG) in the stress response. Steroids.

[66] Herbert Z (2005). Identification of sex hormone-binding globulin in the human hypothalamus. Neuroendocrinology.

[67] Sendemir E, Herbert Z, Caldwell JD, Jirikowski GF (2006). Changes of sex hormone-binding globulin/SHBG expression in the hypothalamo-hypophyseal system of rats during pregnancy, parturition and lactation. Horm. Metab. Res..

[68] Vincze C (2010). Distribution of mRNAs encoding transforming growth factors-β1,-2, and-3 in the intact rat brain and after experimentally induced focal ischemia. J. Comp. Neurol..

[69] Fodor A (2012). Maternal neglect with reduced depressive-like behavior and blunted c-fos activation in Brattleboro mothers, the role of central vasopressin. Horm. Behav..

[70] Cservenák M (2013). Thalamic neuropeptide mediating the effects of nursing on lactation and maternal motivation. Psychoneuroendocrino.

